# OHDLF: A Method for Selecting Orthologous Genes for Phylogenetic Construction and Its Application in the Genus *Camellia*

**DOI:** 10.3390/genes15111404

**Published:** 2024-10-30

**Authors:** Junhao Cai, Cui Lu, Yuwei Cui, Zhentao Wang, Qunjie Zhang

**Affiliations:** 1 Center of Genomics and Bioinformatics, Guangdong Key Laboratory of Plant Molecular Breeding, College of Agriculture, South China Agricultural University, Guangzhou 510642, China; cjh13640310908@163.com (J.C.); threeleben@gmail.com (Y.C.); interestingcn01@gmail.com (Z.W.); 2 Institute of Horticulture, Jiangxi Academy of Agricultural Sciences, Nanchang 330200, China; lucui77@163.com

**Keywords:** phylogenetic tree construction, orthologous genes selection, *Camellia* genus, heterozygosity

## Abstract

Accurate phylogenetic tree construction for species without reference genomes often relies on de novo transcriptome assembly to identify single-copy orthologous genes. However, challenges such as whole-genome duplication (WGD), heterozygosity, gene duplication, and loss can hinder the selection of these genes, leading to limited data for constructing reliable species trees. To address these issues, we developed a new analytical pipeline, OHDLF (Orthologous Haploid Duplication and Loss Filter), which filters orthologous genes from transcript data and adapts parameter settings based on genomic characteristics for further phylogenetic tree construction. In this study, we applied OHDLF to the genus *Camellia* and evaluated its effectiveness in constructing phylogenetic trees. The results highlighted the pipeline’s ability to handle challenges like high heterozygosity and recent gene duplications by selectively retaining genes with a missing rate and merging duplicates with high similarity. This approach ensured the preservation of informative sites and produced a highly supported consensus tree for *Camellia*. Additionally, we evaluate the accuracy of the OHDLF phylogenetic trees for different species, demonstrating that the OHDLF pipeline provides a flexible and effective method for selecting orthologous genes and constructing accurate phylogenetic trees, adapting to the genomic characteristics of various plant groups.

## 1. Introduction

*Camellia* is the largest genus within the Theaceae family and holds significant economic and ecological value, particularly in tea production, where *Camellia sinensis* is widely cultivated for its leaves, making it one of the most important beverage plants globally. Additionally, oil-tea trees produce camellia oil, extracted from the fruit, which is known for its health benefits due to its high content of unsaturated fatty acids. Various *Camellia* species are also valued for their ornamental flowers, further enhancing their importance in both commercial and ecological contexts. Due to polyploidy and extensive hybridization, the phylogenetic relationships within the genus *Camellia* are complex. Chang [1] and Ming [2] proposed influential classification systems based on morphology, cytology, chromosomes, and biogeography. In relatively broad acceptance, Chang’s system comprises four subgenera, 20 sections, and 280 species (we will use this system’s nomenclature in our subsequent work). Recent studies by Wu et al. [3] and Zan et al. [4] utilized high-throughput sequencing, but conflicts in key branch placement persist. Despite employing various methods and data-screening criteria, low support for these branches hinders genus phylogeny resolution.

In plants, conflicts among gene trees are common at various taxonomic levels, with hybridization or gene introgression being the primary causes of the widespread phylogenetic conflicts observed [5,6,7,8]. When analyzing species with reference genomes, the strategy developed by Huang et al. [9] for phasing heterozygous loci can be used to resolve tree conflicts arising from the assembly of single haplotype genomes. For analyzing a group of species with high introgression rate, the phylogenetic tree construction algorithm MIKE, developed by Wang et al. [10] and based on short-read alignments, can be used to address issues of polyploidy and heterozygosity. When constructing species trees from multiple gene trees using orthologous proteins, the two primary methods are the concatenation method and the coalescent method. The concatenation method merges individual gene sequences into a single “supermatrix” for tree construction [11]. Coalescent methods allow for each locus to have its independent evolutionary history, with commonly used algorithms including ASTRAL [12], ASTID [13], and DISTIQUE [14]. When the accuracy of gene phylogenies is low, the concatenation method yields phylogenetic trees with higher support values than the coalescent method [11]. However, constructing large trees consumes significant memory and computation time, and phenomena like gene duplication and loss (GDL) can result in different evolutionary histories for various genomic regions, potentially affecting the accuracy of the tree construction [15,16]. Willson et al. [17] proposed the DISCO pipeline for estimating gene family trees and species trees. When the species under study has a reference genome, genome-guided phylo-transcriptomic methods can be used to construct accurate phylogenetic trees [18]. Low-copy resequencing data can increase the quantity of informative genetic data available, which may aid phylogenetic analysis under appropriate model conditions [19].

The current reference genomes for the genus *Camellia* are primarily focused on tea trees and oil-tea trees, with most species in the genus lacking reference genomes [20,21,22,23,24,25,26,27,28]. The genome sizes of *Camellia* species range between 2.72 to 3.10 Gb, with transposable elements (TEs) comprising 69.00% to 87.41% of the genome. The most recent whole-genome duplication (WGD) event, Ad-β in the *Camellia* genus occurred approximately 100 million years ago [29]. The impact of TE insertions, high heterozygosity, polyploidization, and gene gain and loss may serve as significant contributors to structural changes and gene innovation in this genus [20,30]. Phylogenetic trees of *Camellia* species are typically constructed using transcripts obtained from de novo assembly with Trinity (Wu et al. [3] and Zan et al. [4]), but heterozygosity and recent gene amplification significantly impact the construction of these phylogenetic trees. While various methods aim to improve orthologous gene selection, the distinct evolutionary characteristics of different plant groups may lead to inaccuracies if inappropriate methods are used, hindering a clear understanding of evolutionary processes in the genus *Camellia*.

To address the phylogenetic issues within the genus *Camellia*, we compared the characteristics of heterozygous genomes with those of transcripts assembled de novo using Trinity, which exhibited high heterozygosity along with a large number of gene duplications and losses. Based on these characteristics, we developed the OHDLF (Orthologous Haploid Duplication and Loss Filter), a pipeline specifically designed to filter and merge heterogeneity, duplication, and loss in orthogroups for further phylogenetic tree construction from de novo-assembled transcriptomes. Subsequently, we constructed phylogenetic trees using both coalescent and concatenation methods. Our OHDLF pipeline demonstrated excellent performance in constructing phylogenetic trees using unreferenced transcriptome data from highly heterozygous and complex species, thereby enhancing our understanding of phylogenetic relationships within the genus *Camellia*.

## 2. Materials and Methods

### 2.1. Analysis Data Source

The genus *Camellia* comprises over 200 species [2]. In the phylogenetic analyses conducted by Wu et al. [3], 116 species were included, while Zan et al. [4] analyzed 87 species. The primary difference between these two studies lies in the phylogenetic placement of different clades, although species within the same clade were consistently grouped together. In our study, we selected 2 to 4 representative species from each of the seven major clades within the genus, along with two species from basal lineages, resulting in a total of 24 *Camellia* species for analysis. This selection aims to elucidate the evolutionary relationships between different clades. The subgroups within the genus *Camellia* were named according to the classification by Chang (1998) [2]. When testing the workflow with data at the family level, in addition to the aforementioned 24 *Camellia* species, we also included 14 species from four other genera within the Theaceae family. The names and classifications of these species are provided in Table S1. The data were downloaded from the National Genomics Data Center (NGDC) and the National Center for Biotechnology Information (NCBI) databases (Table S1). Since this study does not aim to classify all species but rather to resolve several major taxonomic discrepancies within the genus *Camellia* and to establish methodological workflows, the species selection criteria were as follows: (1) the phenotype of the species clearly conforms to the descriptions in the flora, (2) the data quality is high and representative, and (3) *Stewartia cordifolia* and *S. sinensis* were selected as outgroups.

To expand the applicability of OHDLF in different species, we also used 9 species representing the genus *Oryza* as a test dataset. The data were downloaded from NCBI databases (Table S2).

### 2.2. Inference of Whole-Genome Duplication

To compare the potential effects of WGDs and heterozygous peaks on orthologous analysis in the hybrid genome of *Camellia*, we analyzed the two haplotypes of genome and transcriptome data of *Camellia crapnelliana* obtained in our laboratory. We used the Python package “wgd” (v2.0) [31] to input the synonymous mutation substitution distribution data (ranging from 0.05 to 3). The command “mcl” was used for sequence alignment and clustering of each CDS, and the commands “ksd” and “mix” were used to construct the *Ks* distribution and *Ks* distribution mixture model. The BGMM method in the “wgd” package was used to analyze the mixture model.

### 2.3. Transcriptome Assembly and Analysis

For the obtained transcriptome data, quality control was performed using Trimmomatic (v0.39) [32]. The quality-controlled reads were de novo-assembled into transcripts using Trinity (v2.15.1) [33]. The assembled transcripts were processed with CD-HIT (v4.8.1) [34], setting the parameter “-c 0.99” to cluster nucleotide sequences with a similarity greater than or equal to 99% in order to remove redundant transcripts generated during the Trinity assembly. Then, TransDecoder (v5.5.0) [33] was used to predict the open reading frames (ORFs) of the filtered nucleotide sequences and translate them into protein sequences. The longest protein sequence from the resulting protein sequence file was extracted as the input file for subsequent analysis.

### 2.4. Pipeline for OHDLF

OrthoFinder (v2.5.4) [35] was used to construct a dataset of homologous genes for the input species. Then, using the OHDLF workflow, orthologous genes were filtered, and phylogenetic trees were constructed using different methods. The specific workflow is as follows:

(1) Select orthologous groups (OGs) with a species missing rate less than or equal to “max_missing_rate” (default 0.05) and overall max duplication number less than or equal to “max_duplication_num” (default 6). Calculate the sequence similarity of multiple copies of the same species in each OG, filter out low-copy orthologous genes with “min_similarity” (default 97%), merge similar sites, and trim divergent sites. Use MAFFT (v7.525) [36] to align the amino acid sequences of the merged low-copy orthologous genes.

(2) If the concatenation method is chosen, the script will output a result file named “final_OrthologsAlign_GDL.phy”, concatenate all processed orthogroups into a single “supersequence” that can be used for phylogenetic tree construction later. In the concatenation method, we use RAxML (v8.2.13) [37] to construct a maximum likelihood (ML) phylogenetic tree. We conducted a rapid bootstrap analysis (1000 replicates) and searched for the best-scoring ML tree simultaneously (the “-f a” option). The GTRGAMMA model was used in this analysis.

(3) If the coalescence method is opted for, the script will output a result file named “all.trees”, which will subsequently be used for tree construction with ASTRAL. In the coalescent method, use IQ-TREE (v2.2.2.3) [38] to construct ML trees for each OG separately with 1000 replicates and search for the best model to the analysis (the “-m MFP” option). Subsequently, use ASTRAL-Pro [39] to reconstruct the coalescent species tree.

We provide an open-source script, OHDLF, which reads the orthogroups generated by OrthoFinder, performs filtering and merging, and outputs files for subsequent phylogenetic tree construction. OHDLF is a standalone command-line program written in Python, runnable on most Linux systems, and is freely available at https://github.com/qunjie-zhang/OHDLF, accessed on 2 August 2024. We have provided an OHDLF.yaml environment configuration package on GitHub. Users can download and directly run the OHDLF script in this environment.

### 2.5. Calculation of Species Divergence Times

R8s (v1.81) [40] was used to create the ultrametric tree. The divergence time between *C. sinensis* and *S. sinensis* was based on previous studies [29,41].

## 3. Results and Discussion

### 3.1. Current Challenges in Using Whole-Genome Transcripts for Accurate Phylogenetic Tree of the Genus Camellia

Incomplete lineage sorting, horizontal gene transfer, introgression, recombination, duplication and loss, and convergent evolution significantly impact the accurate reconstruction of phylogenetic relationships using whole-genome gene trees [5,6,7]. In the studies by Wu et al. [3] and Zan et al. [4], transcriptome sequencing data were assembled to obtain transcript information for constructing phylogenetic trees in the genus *Camellia* using different methods, leading to varying results. The main differences among these methods lie in the way they identify orthologous gene copies, and their tree construction approaches, whether concatenation or coalescent. However, these methods do not consider the genomic characteristics of the *Camellia* species.

**Figure 1:**
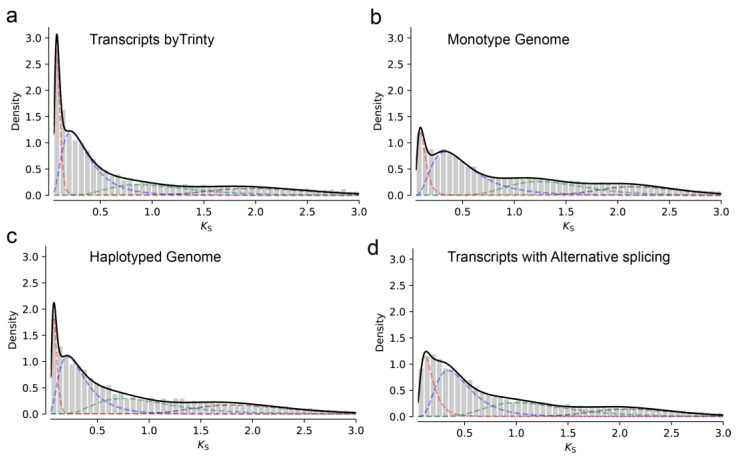
The distribution of synonymous mutation substitutions and the primary sources of highly similar homologous genes in the genus *Camellia*. Synonymous mutation substitution distribution of transcripts from Trinity assembly data (**a**), transcripts from a single haploid genome (**b**), transcripts from a diploid genome with phased haplotypes (**c**), and transcripts with alternative splicing data in the genome (**d**). The red dashed lines represent heterozygous peaks, while the blue and green dashed lines represent the two known rounds of WGDs.

Therefore, we first compared the characteristics of recent gene duplication in de novo-assembled transcripts and various genome assembly annotations from the *C. sinensis* cultivar “Tieguanyin” (TGY) genome to explore the main drivers of gene duplication in the *Camellia* genus [20]. In the de novo [33]-assembled transcriptome, the two rounds of WGDs in the *Camellia* genus are represented by green peaks, both occurring over 100 million years ago [29]. The red peak is attributed to heterozygosity, recent gene duplications, or alternative splicing, posing a challenge for ortholog-based phylogenetic analysis (Figure 1a). To confirm the composition of this peak in *Camellia* species, we analyzed the single haploid genome (Figure 1b), two haplotype genomes (Figure 1c), and genes with variable splicing (Figure 1d). The results indicate that heterozygosity (Figure 1c) is likely the primary obstacle limiting the number of available genes for constructing phylogenetic trees using a 1:1 orthologous gene when analyzing transcript data.

### 3.2. Development of the OHDLF Workflow for Filtering Orthologous Genes in Camellia Species

**Figure 2:**
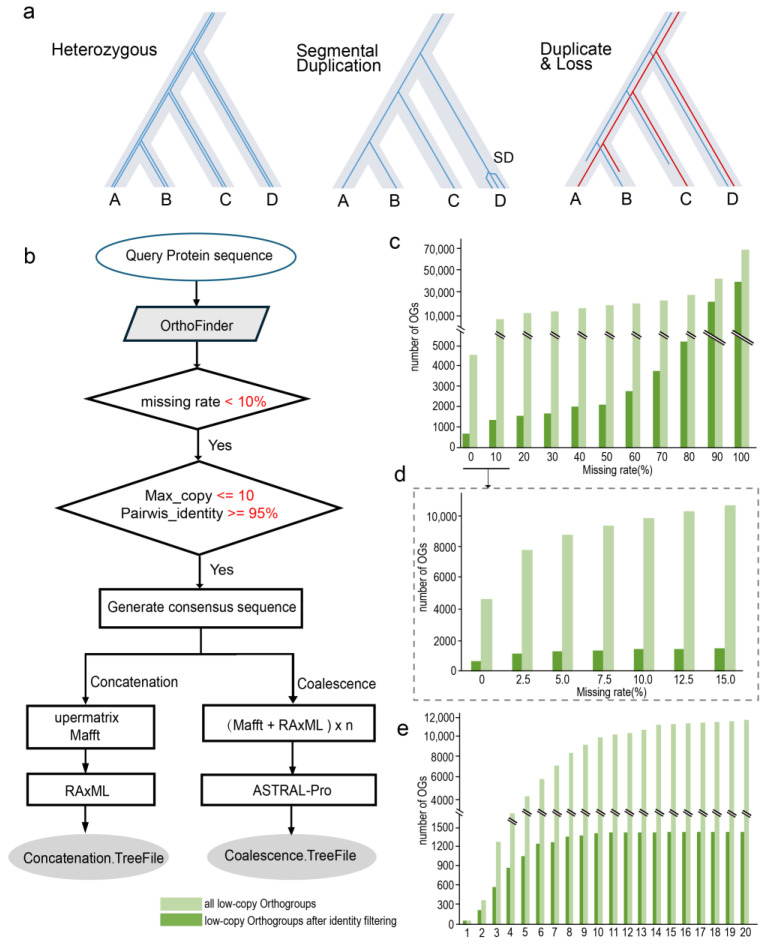
Characteristics of orthogroups and the phylogenetic tree construction process in *Camellia* species. (**a**) The OHDLF pipeline addresses three major issues: heterozygosity, recent segmental duplications, and ancient duplications and losses encountered in the analysis of orthologous genes. (**b**) Detailed steps of the OHDLF process. (**c**,**d**) Data distribution across orthogroups with different mission rates. (**e**) Distribution of the maximum copy number within the same orthogroup in a single species. Light green bars represent the number of orthogroups with different mission rates as identified by OrthoFinder. Dark green bars represent the number of orthogroups meeting the criteria of Max_copy <= 10 and pairwise_identity >= 95%.

Obtaining an accurate phylogenetic tree depends on having sufficient informative loci. A major issue in constructing the phylogeny of the *Camellia* genus lies in the lack of an orthogroup filtering method specifically designed for de novo transcriptomes of genomes with high heterozygosity and high segmental duplication. Based on the genomic characteristics of *Camellia*, we developed a workflow named OHDLF. This workflow is designed to filter out heterozygosity, duplications, and losses in orthologous genes (Figure 2a). The workflow allows for the retention of orthogroups with a certain loss rate, while merging highly similar multi-copy genes within a single species to generate consensus sequences. Subsequently, phylogenetic trees are constructed using both concatenation and coalescent methods (Figure 2b), with detailed steps outlined in the Materials and Methods Section.

We utilized transcriptome data from 24 *Camellia* species and constructed orthogroups using OrthoFinder [35], resulting in a total of 135569 orthogroups. Due to tissue-specific expression of genes, many low-copy orthogroups (copies ≤ 10) exhibit missing genes in individual species. Specifically, when the missing rate is 5%, there are 8757 orthogroups, and when the missing rate is 10%, there are 9842 orthogroups (Figure 2c,d, light green). We then analyzed the multi-copy gene situation, finding only 61 one-to-one orthogroups. As the number of gene copies increases, the number of orthogroups rises rapidly, with 5956 orthogroups having up to 6 copies, with 9842 orthogroups having up to 10 copies (Figure 2e, light green). To merge recently duplicated genes, we combined multi-copy genes with less than 10 copies and more than 95% similarity within orthogroups having a missing rate of less than 10%, producing consensus sequences. Ultimately, 1289 orthogroups were selected for concatenation phylogenetic tree construction. We then adjusted the filtering parameters, lowering the threshold for 10 copies and more than 95% similarity to 6 copies and more than 97% similarity, and 702 orthogroups resulting in our coalescent tree matching our concatenation tree. This demonstrates that data preprocessing using OHDLF can yield higher support values inphylogenetic tree.

### 3.3. Phylogenetic Trees Constructed Using Orthogroup Data from the OHDLF Workflow

**Figure 3:**
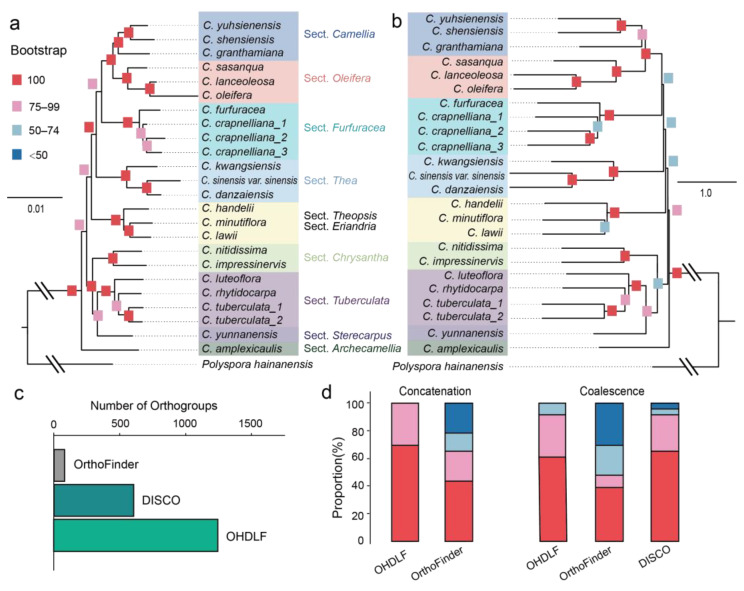
Phylogenetic trees constructed using the OHDLF pipeline. Concatenation (**a**) and coalescent (**b**) phylogenetic trees of *Camellia* species; (**c**) the number of selected orthogroups by OrthoFinder, DISCO [17], and OHDLF can be used for phylogenetic tree construction. (**d**) Bootstrap value distribution of different tree-building methods. Red squares indicate a bootstrap value of 100; pink squares indicate a bootstrap value of 75 to 99; pale blue squares indicate a bootstrap value of 50 to 74; deep blue squares indicate a bootstrap value below 50. The Latin names and grouping information for species used in this figure can be found in Supplemental Table S1.

Using the orthogroups data obtained from the OHDLF workflow (Figure 2b), we constructed both concatenation (Figure 3a) and coalescent (Figure 3b) phylogenetic trees for the genus *Camellia*. Both trees support the same phylogenetic relationships among sections, but the concatenation tree exhibits very high support rates. The phylogenetic position of the Sect. *Furfuracea* and the Sect. *Theopsis* clade (including Sect. *Eriandria*) in both trees supports the findings of concatenation tree by Wu et al. [3], who used concatenation. Our analysis of the Sect. *Chrysantha* and Sect. *Tuberculata* branching relationships supports the coalescent trees of Wu et al. [3] and Zan et al. [4]. The concatenation tree constructed using orthogroups from the OHDLF workflow provides a highly supported and consistent tree, with all branch bootstrap values above 80 (Figure 3a). In contrast, the coalescent tree may show lower support rates due to regions of ILS and introgression; these branch points could serve as focal points for future studies on population evolution.

Subsequently, we further compared the orthogroup number obtained using DISCO [17] and OHDLF from OrthoFinder, as well as the branch support values after tree construction (Figure 3c,d). The results showed that the orthogroups filtered by DISCO and OHDLF using default parameters were 602 and 1289, respectively (Figure 3c). The coalescent tree constructed with DISCO showed results consistent with those of Zan et al. [4], where 13.5% of nodes had support values below 85% (Figure 3d). These key nodes are the source of differences observed in the studies by Wu et al. [3] and Zan et al. [4].

In contrast, the concatenation tree constructed by OHDLF showed all bootstrap values above 85%, providing a clear view of the species’ evolutionary background (Figure 3d). To further validate these results, a comparison with the coalescent tree was performed. For branches with low support in the parallel trees, the “all.trees” output from the OHDLF pipeline’s coalescent methods could employed to elucidate the reticulate evolutionary relationships in highly heterozygous species [20].

### 3.4. The Accuracy of Phylogenetic Tree Construction Using the OHDLF Pipeline and Its Scope of Application

**Figure 4:**
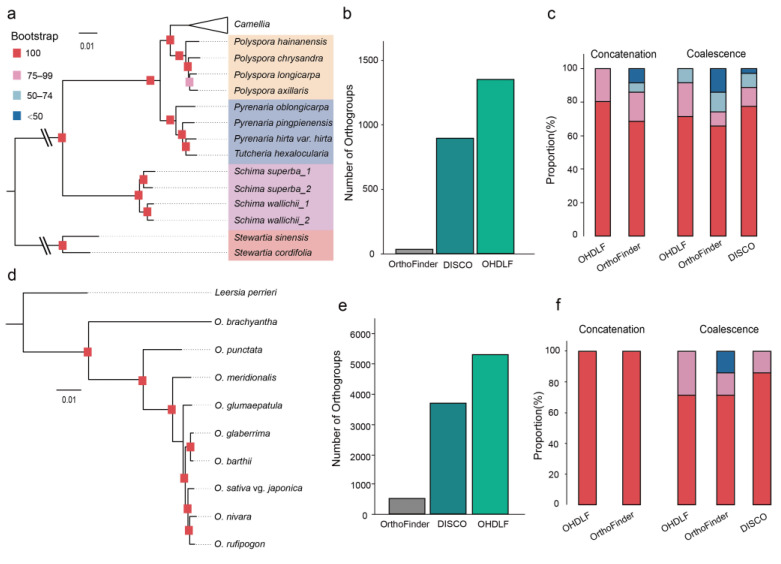
A comparison of OHDLF usage across different species. (**a**) Phylogenetic tree of family Theaceae. The number of selected orthogroups (**b**) and bootstrap value distribution (**c**) by OrthoFinder and DISCO for the phylogenetic tree of family Theaceae. (**d**) Concatenation phylogenetic trees of *Oryza* species. The number of selected orthogroups (**e**) and bootstrap value distribution (**f**) comparison for the phylogenetic tree of genus *Oryza*. Red squares indicate a bootstrap value of 100; pink squares indicate a bootstrap value of 75 to 99; pale blue squares indicate a bootstrap value of 50 to 74; deep blue squares indicate a bootstrap value below 50 for (**a**,**c**,**d**,**f**). The Latin names and data source for the species used in (**a**,**d**) can be found in Supplemental Tables S1 and S2.

After demonstrating the effectiveness and accuracy of OHDLF for constructing phylogenetic trees in the genus *Camellia*, we further explored the pipeline’s applicability. We first examined the results at the family level by constructing phylogenetic trees for Theaceae species, incorporating 14 species from four genera (*Polyspora*, *Pyrenaria*, *Schima*, and *Stewartia*) for testing (Figure 4a). Among the 38 species in the Theaceae family analyzed using transcriptomes, only 27 single-copy orthogroups were identified using OrthoFinder. The orthogroups filtered by DISCO and OHDLF with default parameters yielded 871 and 1366 orthogroups (Figure 4b), respectively. Notably, all bootstrap values in the OHDLF coalescent trees exceeded 80%, and bootstrap values in the concatenation trees were above 60%, while 2.86% of the bootstrap values in DISCO’s concatenation trees were below 50% (Figure 4c). The concatenation (Figure 4a) and coalescent (Supplemental Figure S1) trees produced by OHDLF exhibited identical phylogenetic relationships, aligning with the findings of Zhang et al. [29], who utilized resequencing and RNA-seq data. This indicates that the OHDLF method is also applicable to Theaceae.

Subsequently, we compared the results using the monocot *Oryza* genus. We included nine species from the *Oryza* genus with clear phylogenetic relationships and *Lecomtella perrieri* as an outgroup (Figure 4d) [42]. Using input from unreferenced transcriptomes assembled from a single tissue, both DISCO and OHDLF yielded results consistent with the whole-genome phylogenetic analyses conducted by Stein et al. [42]. Given the low genomic complexity of rice, OrthoFinder identified 531 single-copy orthogroups, while the orthogroups filtered by DISCO and OHDLF were 3675 and 5289, respectively (Figure 4e). The coalescent analysis using OHDLF showed bootstrap values of 100%, while both DISCO and OHDLF concatenation trees had values exceeding 80% (Figure 4f). This result suggests that DISCO and OHDLF perform comparably in species with lower genomic complexity.

It is important to note that all species selected in this analysis were non-hybrid polyploids, and the OHDLF method was not developed to account for allopolyploidy. Meanwhile, when using OHDLF, analyzing the genomic characteristics of the target species first and adjusting the parameters according to the data features can result in a higher support value phylogenetic tree.

### 3.5. The Time Tree of Family Theaceae

**Figure 5:**
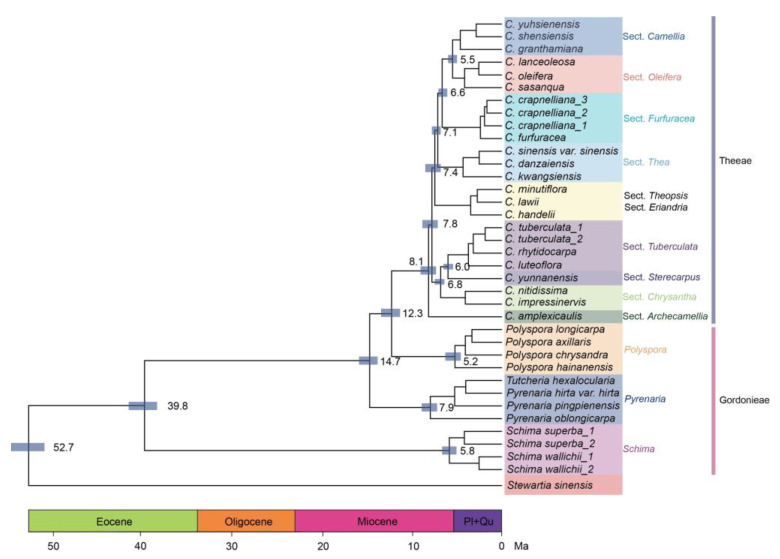
The time tree of family Theaceae. Ages of stratigraphic boundaries were from the Int. Chronostrat. Chart [43] (Pl: Pliocene; Qu: Quaternary), in millions of years ago (Mya). Blue bars at each node show the 95% highest posterior density (HPD) with posterior probability > 0.5.

After obtaining an accurate method for constructing the phylogenetic tree, we recalculated the divergence times of various groups within the Theaceae family. We used *S. sinensis* from the tribe *Stewartieae* as the outgroup. Based on previous studies on divergence times [29,41], we recalculated the phylogenetic divergence times. The most recent common ancestor (MRCA) of the Theaceae family is estimated to have diverged around 52.7 Mya, during the Eocene epoch. Theeae and Gordonieae were also recovered as sisters. The divergence of tribes Theeae and Gordonieae occurred approximately 39.8 Mya, also during the Eocene epoch. The crown ages of Gordonieae and Theeae were estimated to be 5.8 Mya and 14.7 Mya, respectively. Within the tribe Theeae, *Camellia* and *Polyspora* are sister groups, diverging at around 12.3 Mya (Figure 5).

Then, there was a rapid diversification of species in the genus *Camellia* after 8.1 Mya, with significant overlap in the divergence times of groups such as the Sect. *Camellia*, Sect. *Oleifera*, and Sect. *Furfuracea* (Figure 5). This is due to the extensive overlap in the distribution areas of these groups, where there was a large amount of interspecific hybridization in the early stages of population differentiation, and polyploidization events occurred, making it more complex to determine the evolutionary relationships between different groups [20,30]. Therefore, when constructing the phylogenetic relationships of the genus *Camellia*, it is necessary to set parameters according to the evolutionary characteristics of the genus genome to obtain an accurate phylogenetic tree.

## 4. Conclusions

In conclusion, the OHDLF pipeline offers a robust method for constructing phylogenetic trees for species without reference genomes. It effectively addresses challenges like whole-genome duplication and heterozygosity by selectively retaining single-copy orthologous genes, resulting in a highly supported consensus tree for *Camellia*.

Building on the concatenated tree with high support values, we further applied the coalescent method within the OHDLF pipeline to perform parallel tree analysis. This combined approach allows for deeper insights into the phylogenetic relationships of highly heterozygous species, which are often difficult to resolve using a single method alone.

However, the current design of OHDLF may limit its applicability for analyzing allopolyploid species, highlighting the need for future adaptations. Overall, this pipeline enhances the reliability of phylogenetic analyses and offers a flexible tool for studying diverse plant groups, while also underscoring the importance of tailored approaches for more complex genomic scenarios.

## Data Availability

The OHDLF is accessible at https://github.com/qunjie-zhang/OHDLF, accessed on 2 August 2024.

## Supplementary Materials

The following supporting information can be downloaded at: https://www.mdpi.com/article/10.3390/genes15111404/s1, Table S1: Summary of the RNA sequencing data of *Camellia* plants and its close species. Table S2: Summary of the RNA sequencing data of *Oryza* plants and its close species. Figure S1: Coalescent phylogenetic tree of Theaceae species.
