# Supplementary material for: OHDLF: A Method for Selecting Orthologous Genes for Phylogenetic Construction and Its Application in the Genus Camellia
Source: Genes (Basel). 2024 Oct 30;15(11):1404. doi: 10.3390/genes15111404 (PMC11593501; doi:10.3390/genes15111404)
Supplement: Supplementary file 1 [file genes-15-01404-s001.zip › Supplementary Figure S1.pdf]

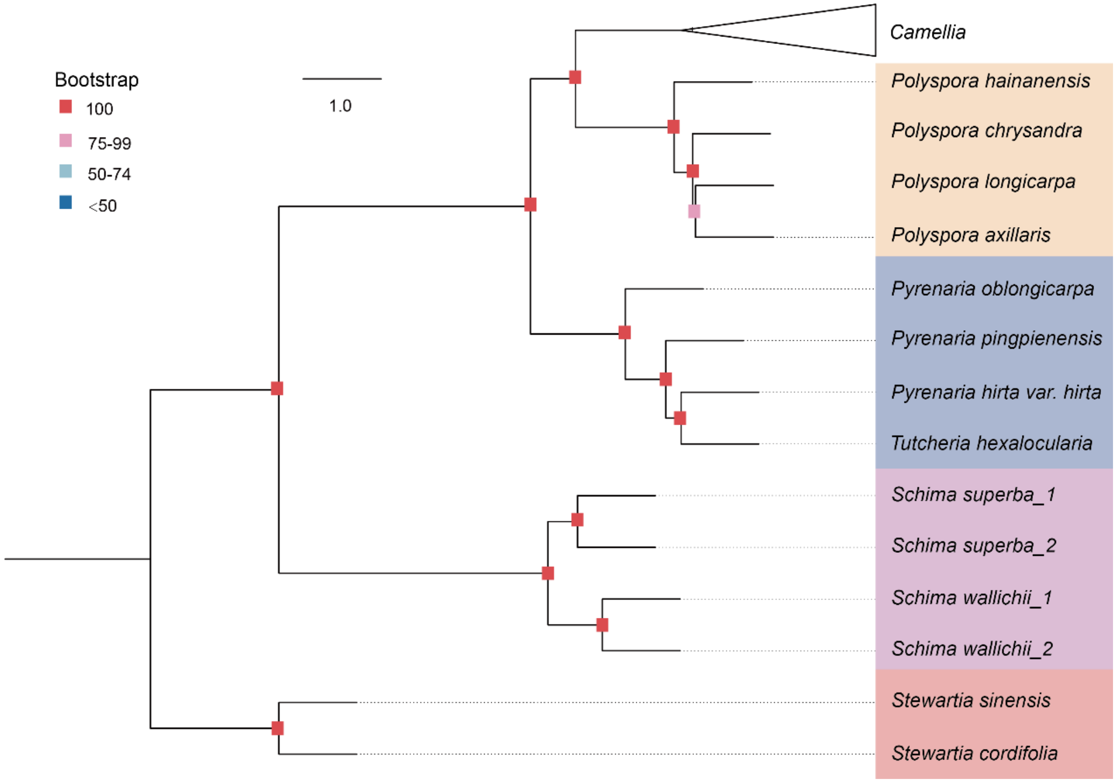

Figure S1 Coalescent phylogenetic tree of Theaceae species. Red squares indicate a bootstrap value of 100; pink squares indicate a bootstrap value of 75 to 99; pale blue squares indicate a bootstrap value of 50 to 74; deep blue squares indicate a bootstrap value below 50.
